# *ZBTB12* DNA methylation is associated with coagulation- and inflammation-related blood cell parameters: findings from the Moli-family cohort

**DOI:** 10.1186/s13148-019-0665-6

**Published:** 2019-05-10

**Authors:** Fabrizia Noro, Francesco Gianfagna, Alessandro Gialluisi, Amalia De Curtis, Augusto Di Castelnuovo, Emanuela Napoleone, Chiara Cerletti, Maria Benedetta Donati, Giovanni de Gaetano, Marc F. Hoylaerts, Licia Iacoviello, Benedetta Izzi, on behalf of the Moli-family study Investigators, Licia Iacoviello, Branislav Vohnout, Marcello Arca, Chiara Cerletti, Maria Benedetta Donati, Giovanni de Gaetano, Roberto Lorenzet, Augusto di Castelnuovo, Simona Costanzo, Francesco Gianfagna, Romina di Giuseppe, Antonella Cutrone, Amalia De Curtis, Sara Magnacca, Benedetta Izzi, Marilena Crescente, Agnieszka Pampuch, Chiara Tamburrelli, Emanuela Napoleone, Filomena Zurlo, Luisa Nanni

**Affiliations:** 1 Department of Epidemiology and Prevention, IRCCS NEUROMED, Pozzilli, IS Italy; 2 Department of Medicine and Surgery, University of Insubria, Varese, Italy; 3 Mediterranea Cardiocentro, Naples, Italy; 4 Department of Cardiovascular Sciences, Center for Molecular and Vascular Biology, University of Leuven, Leuven, Belgium; 5 Present address: Viale del Cimitero 20, 66054 Vasto, CH Italy

**Keywords:** DNA methylation, Granulocyte counts, White blood cell counts, Whole blood coagulation time, Zinc fingers, Cardiovascular risk

## Abstract

**Background:**

*Zinc finger and BTB domain-containing protein 12* (*ZBTB12*) is a predicted transcription factor with potential role in hematopoietic development. Recent evidence linked low methylation level of *ZBTB12* exon1 to myocardial infarction (MI) risk. However, the role of *ZBTB12* in the pathogenesis of MI and cardiovascular disease in general is not yet clarified. We investigated the relation between *ZBTB12* methylation and several blood parameters related to cardio-cerebrovascular risk in an Italian family-based cohort.

**Results:**

*ZBTB12* methylation was analyzed on white blood cells from the Moli-family cohort using the Sequenom EpiTYPER MassARRAY (Agena). A total of 13 CpG Sequenom units were analyzed in the small CpG island located in the only translated *ZBTB12* exon. Principal component analysis (PCA) was performed to identify groups of CpG units with similar methylation estimates. Linear mixed effect regressions showed a positive association between methylation of *ZBTB12* Factor 2 (including CpG units 8, 9–10, 16, 21) and TNF-ɑ stimulated procoagulant activity, a measure of procoagulant and inflammatory potential of blood cells. In addition, we also found a negative association between methylation of *ZBTB12* Factor 1 (mainly characterized by CpG units 1, 3–4, 5, 11, and 26) and white blood cell and granulocyte counts. An in silico prediction analysis identified granulopoiesis- and hematopoiesis-specific transcription factors to potentially bind DNA sequences encompassing CpG1, CpG3–4, and CpG11.

**Conclusions:**

*ZBTB12* hypomethylation is linked to shorter TNF-ɑ stimulated whole blood coagulation time and increased WBC and granulocyte counts, further elucidating the possible link between *ZBTB12* methylation and cardiovascular disease risk.

**Electronic supplementary material:**

The online version of this article (10.1186/s13148-019-0665-6) contains supplementary material, which is available to authorized users.

## Background

The *zinc finger and BTB domain-containing protein 12* (*ZBTB12*) is a predicted transcription factor belonging to the big family of methyl-CpG-binding proteins (MBPs) [1]. ZBTB12 consists of four C-terminal C2H2/Krüppel-type zinc finger domains predicted to bind to DNA, and an N-terminal BTB (broad-complex, tram-track, and bric-a-brac) domain for protein–protein interactions [2]. ZBTB proteins are described to play a role in hematopoietic development, differentiation and lineage fate determination [3], and malignant transformation [4]. Guarrera and colleagues [5] performed a genome-wide DNA methylation analysis in white blood cells (WBC) from two European cohorts and identified a region in *ZBTB12* as the top differentially methylated genomic region in patients with myocardial infarction (MI) [5]. *ZBTB12* hypomethylation was associated with MI risk, and the association was more pronounced in cases with shorter time to disease [5]. Despite *ZBTB12* is expressed in most human tissues (Human Protein Atlas available from www.proteinatlas.org), its function and possible role in MI pathogenesis are still unknown.

In light of *ZBTB12*’s potential role in hematopoiesis and MI risk, we investigated the association between *ZBTB12* methylation patterns in the Moli-family cohort [6] and different blood cell parameters related to coagulation, inflammation, and cardiovascular disease (CVD) risk including whole blood clotting time, platelet–leukocyte mixed aggregates, and blood cell counts, previously suggested as CVD risk factors [6–10].

## Results

**Fig. 1:**
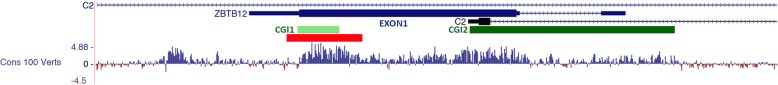
*ZBTB12* structure (chr6:31899617-31901992, GRCh38/hg38 Assembly). Exon1 is indicated by a full blue box (“EXON1”). Two CpG islands are located in the gene (“CGI1” and “CGI2,” depicted as light and dark green boxes, respectively). CpG islands are defined based on the formula described by Gardiner–Garden et al. *J Mol Biol*. 1987;196(2):261–282. *ZBTB12* conservation across vertebrates is displayed as blue histograms at the bottom of the figure using the Vertebrate Multiz Alignment & Conservation (100 Species) UCSC track. Sequenom studied region (chr6: 31899847-31900326, GRCh38/hg38 Assembly) is depicted as red box

**Fig. 2:**
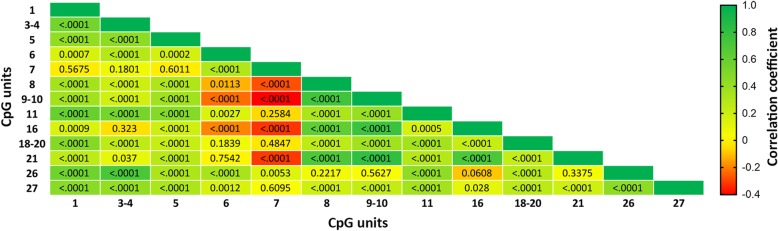
Correlations among the *ZBTB12* CpG units. Heatmap showing *ZBTB12* CpG unit inter-correlations. Correlation coefficient is depicted for each CpG unit pair as color range from red (*r* = − 0.40) up to green (*r* = 1). *P* values of correlations are indicated for each CpG unit pair in the correspondent box

*ZBTB12* is located on chromosome 6 and contains two CpG islands, both covering the only translated exon of the gene (EXON1, Fig. 1). Mean and standard deviation (SD) of methylation levels at the 13 *ZBTB12* units studied are shown in Table 1. To identify possible connections among the *ZBTB12* methylation units studied in the Moli-family cohort, we run a correlation analysis among all CpG units included in the study (Fig. 2). Since we found significant CpG unit inter-correlations, we conducted a principal component analysis (PCA) aiming at identifying common underlying components that could explain the largest part of methylation variability shared across units. Two main methylation factors emerged with PCA (Table 1), explaining a large part of gene methylation variance (86.1%). Factor 1 was characterized by high positive loadings of CpGs 3–4, 26, 1, 11, 5, 27, 18–20, and 6, and Factor 2 showed high loadings of CpGs 9–10, 21, 16, and 8 (Table 1). We first studied the association of *ZBTB12* methylation factors with a number of classical CVD risk factors, including physical activity, smoke, hypertension, dyslipidemia, obesity, diabetes, and alcohol consumption. General characteristics and CVD risk factor distribution in the analyzed cohort are reported in Table 2. By studying the association between *ZBTB12* methylation and these environmental factors, we observed that alcohol intake greater than 15 g/day (*β* = − 0.415, *p* = 0.0024, pFDR significant) was associated with Factor 2, while obesity (*β* = 0.40, *p* = 0.0053) and leisure-time physical activity (− 0.155, *p* = 0.0050) were associated with Factor 1 only with nominal significance (Table 2).

**Table 1:** Distribution of *ZBTB12* factor loadings (*N* = 342) and specific CpG unit methylation in the Moli-family cohort

CpG number	Factor loading		Methylation levels		
	Factor 1	Factor 2	*N*	Mean	SD
3–4	*0.83*	0.03	440	0.37	0.09
26	*0.80*	− 0.04	453	0.37	0.10
1	*0.74*	0.16	458	0.35	0.10
11	*0.70*	0.20	415	0.28	0.14
5	*0.58*	0.19	419	0.11	0.09
27	*0.56*	0.10	408	0.43	0.14
18–19–20	*0.47*	0.12	411	0.71	0.12
6	*0.43*	− 0.15	450	0.64	0.20
9–10	0.27	*0.89*	457	0.24	0.07
21	0.23	*0.83*	458	0.09	0.06
16	0.01	*0.80*	458	0.17	0.10
8	0.26	*0.78*	457	0.06	0.03
7	0.11	− 0.34	421	0.63	0.14

PCA resulted in the identification of two factors with eigenvalue > 1. Factor loadings of the main sites for each factor are highlighted in italics

**Table 2:** Association between *ZBTB12* methylation factors and CVD risk factors

CVD risk factors				Associations between methylation factors and phenotypes						
					Factor 1			Factor 2		
	*N*	Mean	SD	*N*	Beta	SE	*p*-value	Beta	SE	*p* value
Age (years)	458	42.80	18.83	342	0.005	0.003	0.696	− 0.001	0.003	0.806
Leisure-time physical activity (MET/day)	449	2.31	1.07	336	− 0.111	0.052	*0.035*	− 0.066	0.052	0.206
	*N*	*n*	%		Delta	SD	*p* value	Delta	SD	*p* value
Males	458	236	53.7%	342	0.031	0.105	0.768	− 0.148	0.104	0.157
Ever smokers	458	212	46.2%	342	0.182	0.115	0.113	− 0.024	0.114	0.831
Alcohol (> 15 g/day)	422	93	22.0%	319	− 0.013	0.139	0.925	− 0.415	0.135	0.0024*
Hypertension	456	162	35.5%	341	0.040	0.146	0.783	− 0.186	0.144	0.198
Dyslipidemia	456	191	41.9%	341	0.179	0.116	0.125	− 0.150	0.115	0.193
Obesity	456	93	20.4%	341	0.298	0.145	*0.041*	0.165	0.145	0.256
Diabetes	458	26	5.7%	342	0.159	0.232	0.495	− 0.003	0.230	0.988

Model adjusted by age and gender as fixed effects and family stratification as a random effect. Significant *p* values are shown in italics

*MET* metabolic equivalent of task

*pFDR significant (alcohol, pFDR = 0.043)

Then, we used linear mixed effect regression models to evaluate associations between *ZBTB12* methylation and different blood parameters related to coagulation, inflammation, and CVD risk, namely unstimulated and TNFɑ-stimulated coagulation time (along with the resulting unstimulated–stimulated delta difference), platelet–monocyte and platelet–PMN aggregates, and blood cell counts (see Table 3). We did this through a double approach, by investigating association with methylation factors and with single CpG units. Because among the environmental variables associated with *ZBTB12* methylation, only alcohol and obesity were associated with blood cell counts at *p* < 0.1 (data not shown), these variables were additionally included in the model as covariates to study the association between *ZBTB12* methylation and blood cell counts (Tables 3 and 5).

**Table 3:** Association between *ZBTB12* methylation factors and blood cell parameters

	*N*	Mean	SD	Associations between methylation factors and phenotypes						
				*N*	Factor 1			Factor 2		
					Beta	SE	*p*	Beta	SE	*p*
Functions										
Coagulation time (sec.)	417	395.51	77.66	313	− 0.007	0.052	0.891	0.051	0.052	0.333
TNFɑ-stim. coagulation time (sec.)	417	350.90	72.89	313	− 0.021	0.056	0.709	0.160	0.056	0.0047*
Delta coag. time (basal-TNF) (sec.)	417	44.60	56.53	313	− 0.034	0.052	0.510	0.145	0.052	0.0053*
Platelet-monocyte aggr. (%)	450	7.81	9.06	337	0.107	0.045	*0.019*	0.005	0.046	0.912
Platelet-PMN aggr. (%)	449	4.43	4.97	336	0.032	0.049	0.509	− 0.016	0.050	0.743
Blood cell count										
White blood cells (10^9^/L)	458	6.38	1.48	318	− 0.161	0.054	0.0032*	− 0.036	0.055	0.509
Lymphocyte (10^9^/L)	458	2.00	0.58	318	− 0.065	0.056	0.254	− 0.063	0.057	0.271
Monocytes (10^9^/L)	458	0.42	0.17	318	− 0.076	0.052	0.147	0.023	0.053	0.661
Granulocytes (10^9^/L)	458	3.96	1.17	318	− 0.158	0.056	0.0048*	− 0.032	0.056	0.567
Platelets (10^9^/L)	458	253.82	61.84	318	− 0.050	0.052	0.335	− 0.044	0.053	0.407
Red blood cells (10^9^/L)	458	4.91	0.51	318	− 0.046	0.049	0.352	0.003	0.049	0.945

Model adjusted by age, gender, and smoking as fixed effects and family stratification as a random effect; additional covariates were added to the model and were associated to both methylation factors and phenotypes (for blood cell counts, alcohol and obesity). Standardized values of phenotypes and methylation are reported (beta values reported as standard deviation units). *Delta coag. time* is obtained by the difference between unstimulated and TNFɑ-stimulated coagulation time. Significant *p* values are shown in italics

*pFDR significant

**Fig. 3:**
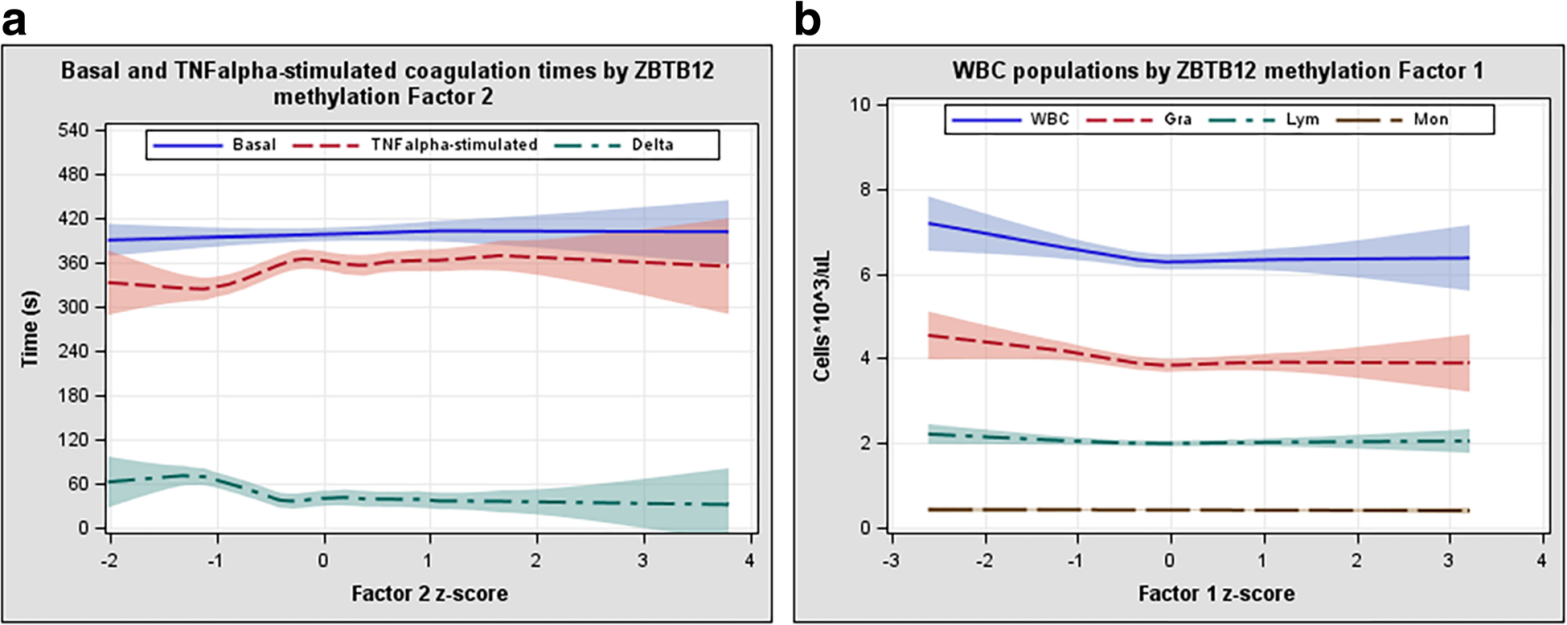
Whole blood clotting times and white blood cell counts by Factor methylation levels. **a** Whole blood clotting times by Factor 2 methylation levels: basal (blue, solid line) and TNF-ɑ-stimulated (red, dashed line) whole blood coagulation times and their difference (Delta = basal minus stimulated; green, dash-dot line). **b** Count of white blood cell (WBC) populations by Factor 1 methylation levels: WBC (blue, solid line) and sub-populations of granulocytes (red, short-dashed line), lymphocytes (green, dot-dashed line), and monocyte (brown, long-dashed line). A local regression with a scatterplot smoothing method that automatically determines the optimal smoothing parameter was used (PROC SGPLOT with LOESS statement in SAS). Local regression method implies that statistical power decreases at extreme *x* values (larger confidence intervals)

We found a significant association between Factor 2 and TNF-α-stimulated whole blood clotting time, with 16.0% increase in SD of clotting time for an increase of 1 SD in Factor 2 (*β* = 0.160, *p* = 0.0047; Table 3). The linear association between TNF-α-stimulated whole blood clotting time and Factor 2 was evident below the median (− 0.15) of adherence to the factor (Fig. 3a). Subjects with low methylation levels at factor units showed a coagulation time reduced by about half a minute (Δ-time in Fig. 3a) compared to those with higher methylation levels. The results did not significantly change when blood cell counts were added to the models as covariates.

Among blood cell count association results, WBC were associated with Factor 1, an association mainly driven by granulocytes (Table 3). In a similar fashion as for Factor 2, this association was evident at adherence to Factor 1 below the median (− 0.05), after which a plateau was reached (Fig. 3b).

A detailed single CpG unit analysis supported the associations observed with methylation factors, reporting a significant positive association (pFDR < 0.05) between Factor 2 single CpG units (CpG8, 9–10, and 16) and TNF-α-stimulated whole blood clotting time (Table 4). When adjusting these associations for blood cell counts, the results did not change.

**Table 4:** Association between *ZBTB12* CpG-specific methylation and blood cell functional parameters

Factor n.	CpG n.	Coagulation time			TNFɑ-stim. coagulation time			Delta coag. time (basal-TNF)			Platelet–monocyte aggregates			Platelet–PMN aggregates		
		Beta	SE	*p*	Beta	SE	*p*	Beta	SE	*p*	Beta	SE	*p*	Beta	SE	*p*
F1	3–4	0.018	0.045	0.690	− 0.019	0.050	0.711	− 0.040	0.049	0.411	0.074	0.039	0.061	0.006	0.043	0.898
	26	− 0.007	0.044	0.877	− 0.052	0.049	0.286	− 0.062	0.048	0.201	0.097	0.039	*0.013*	− 0.003	0.043	0.942
	1	0.023	0.045	0.608	0.038	0.049	0.447	− 0.002	0.048	0.974	0.055	0.039	0.164	− 0.023	0.043	0.602
	11	0.109	0.046	*0.018*	0.074	0.051	0.148	− 0.063	0.051	0.218	0.016	0.041	0.703	− 0.016	0.046	0.735
	5	− 0.031	0.046	0.501	0.013	0.050	0.798	0.066	0.049	0.179	0.068	0.042	0.102	0.067	0.046	0.142
	27	− 0.040	0.047	0.402	− 0.017	0.052	0.743	0.020	0.052	0.704	0.087	0.042	*0.040*	0.041	0.046	0.366
	18–20	0.033	0.044	0.461	0.045	0.048	0.353	− 0.005	0.048	0.912	0.037	0.039	0.342	0.030	0.044	0.496
	6	− 0.043	0.044	0.331	− 0.063	0.048	0.191	− 0.004	0.047	0.932	0.026	0.039	0.502	− 0.052	0.043	0.227
F2	9–10	0.104	0.044	*0.018*	0.154	0.048	0.002*	0.052	0.048	0.274	0.028	0.039	0.478	0.003	0.043	0.951
	21	0.030	0.046	0.505	0.045	0.050	0.368	0.014	0.049	0.771	0.036	0.040	0.369	− 0.008	0.044	0.863
	16	0.052	0.043	0.224	0.134	0.047	0.005*	0.106	0.046	*0.023*	− 0.012	0.038	0.757	− 0.010	0.043	0.820
	8	0.075	0.044	0.091	0.126	0.048	0.010*	0.061	0.047	0.197	0.031	0.040	0.438	0.019	0.044	0.669
	7^§^	− 0.008	0.046	0.859	− 0.069	0.050	0.169	− 0.086	0.046	0.062	− 0.050	0.041	0.230	− 0.039	0.044	0.380

Model adjusted by age, gender, and smoking as fixed effects and family stratification as a random effect; additional covariates were added to the model and were associated to both methylation factors and phenotypes (for blood cell counts, alcohol, and obesity). Standardized values of phenotypes and methylation are reported (beta values reported as standard deviation units). Significant *p* values are shown in italics

*pFDR significant

^§^Factor loading for both Factor 1 and 2 lower than 0.40

Furthermore, we observed significant inverse association between Factor 1 single CpG units and different blood cell counts, including WBC (with CpG1, 3–4, 5, 26, and 11; *p* ≤ 0.018) and granulocytes counts (with CpG1, 3–4, 5, 26, and 27; *p* ≤ 0.007) (Table 5).

**Table 5:** Association between *ZBTB12* CpG-specific methylation and blood cell counts

Factor no.	CpG no.	White blood cells			Lymphocytes			Monocytes			Granulocytes			Platelets			Red blood cells		
		Beta	SE	*p*	Beta	SE	*p*	Beta	SE	*p*	Beta	SE	*p*	Beta	SE	*p*	Beta	SE	*p*
F1	3–4	− 0.110	0.046	0.018*	0.007	0.047	0.879	0.002	0.043	0.972	− 0.143	0.048	0.003*	− 0.030	0.043	0.492	0.033	0.040	0.409
	26	− 0.139	0.045	0.002*	− 0.011	0.047	0.809	− 0.049	0.042	0.253	− 0.166	0.046	0.0004*	− 0.086	0.042	*0.042*	− 0.003	0.039	0.942
	1	− 0.115	0.046	0.013*	− 0.009	0.048	0.854	0.015	0.044	0.736	− 0.143	0.048	0.003*	− 0.062	0.044	0.166	− 0.036	0.040	0.373
	11	− 0.120	0.050	0.016*	− 0.098	0.052	0.059	− 0.018	0.049	0.710	− 0.101	0.051	0.050	0.007	0.047	0.885	0.004	0.044	0.936
	5	− 0.116	0.048	0.016*	0.039	0.050	0.434	− 0.056	0.046	0.219	− 0.151	0.050	0.003*	− 0.037	0.045	0.406	− 0.003	0.042	0.947
	27	− 0.109	0.048	*0.023*	− 0.002	0.050	0.967	− 0.007	0.046	0.884	− 0.134	0.050	0.007*	− 0.012	0.046	0.801	− 0.015	0.043	0.728
	18–20	− 0.012	0.048	0.808	− 0.130	0.049	*0.009*	− 0.059	0.045	0.190	0.053	0.049	0.281	0.008	0.045	0.866	− 0.076	0.042	0.073
	6	0.006	0.047	0.903	0.020	0.048	0.672	− 0.031	0.044	0.485	0.002	0.048	0.960	0.042	0.044	0.341	0.089	0.040	*0.027*
F2	9–10	− 0.049	0.046	0.291	− 0.026	0.048	0.591	0.027	0.044	0.540	− 0.058	0.048	0.230	− 0.036	0.044	0.413	− 0.006	0.040	0.880
	21	− 0.014	0.047	0.766	0.009	0.049	0.856	0.011	0.045	0.814	− 0.029	0.048	0.551	− 0.050	0.045	0.264	− 0.034	0.040	0.400
	16	− 0.015	0.046	0.746	− 0.048	0.047	0.314	0.034	0.043	0.434	− 0.006	0.048	0.901	− 0.011	0.043	0.793	0.031	0.040	0.437
	8	− 0.055	0.047	0.234	− 0.015	0.048	0.763	0.029	0.044	0.510	− 0.071	0.048	0.142	− 0.004	0.045	0.928	− 0.059	0.040	0.145
	7^§^	− 0.056	0.048	0.238	− 0.076	0.049	0.119	− 0.067	0.044	0.128	− 0.028	0.049	0.576	− 0.013	0.046	0.784	0.004	0.041	0.920

Model adjusted by age, gender, and smoking as fixed effects, and family stratification as a random effect; additional covariates were added to the model and were associated to both methylation factors and phenotypes (for blood cell counts, alcohol, and obesity). Standardized values of phenotypes and methylation are reported (beta values reported as standard deviation units). Significant *p* values are shown in italics

*pFDR significant

^§^Factor loading for both Factor 1 and 2 lower than 0.40

**Fig. 4:**
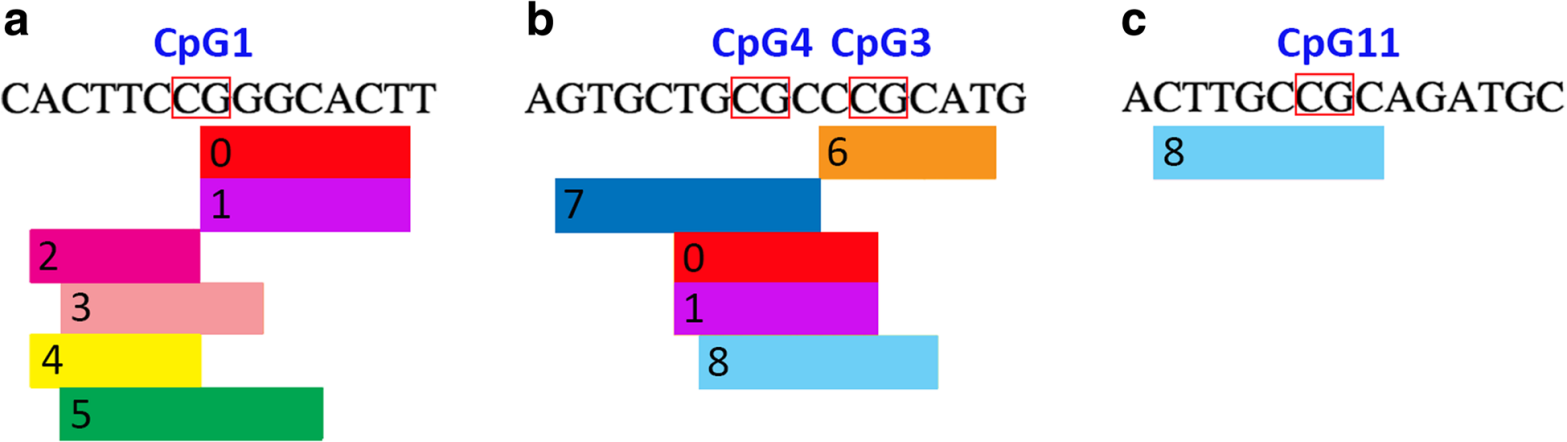
Prediction binding site analysis of *ZBTB12* transcription factors. PROMO/TRANSFAC Transcription Factor Prediction analysis on *ZBTB12* sequences including **a** CpG1, **b** CpG3–4, **c** CpG11. The length of each box indicating the transcription factor identifies its predicted binding sequence. The number into each box identifies the specific transcription factor (0 = PAX-5; 1 = p53; 2 = TFII-I; 3 = c-Ets-1; 4 = STAT4; 5 = Elk-1; 6 = XBP-1; 7 = GCF; 8 = E2F-1)

DNA methylation changes at even only one CpG site can affect transcription factor (TF) binding to the DNA, influencing gene expression. Therefore, we searched for TF putative binding sites encompassing the WBC significantly associated *ZBTB12* CpG sites. We found several TFs predicted to bind CpG units 1, 3–4, 5, 11, 26, and 27 (Additional file 1). Interestingly, among the putative transcription factors identified, we observed a high predictive binding score of PAX-5 and p53 on both CpG units 1 and 3–4, and of E2F-1 on both CpG units 3–4 and 11 (Fig. 4).

## Discussion

Our study shows that the *ZBTB12* methylation profile is associated with whole blood coagulation time after TNF-ɑ stimulation and with WBC and granulocyte counts.

*ZBTB12* is a highly conserved gene among species, but still poorly investigated. Recently, its hypomethylation has been associated with MI risk, in two European cohorts by Guarrera and colleagues [5]. In our study, we identified common linking patterns of the 13 *ZBTB12* CpG units investigated (Factor 1 and Factor 2 in Table 1) that independently affect different CVD-related blood cell characteristics.

On the one hand, *ZBTB12* Factor 2 was significantly associated with both the TNF-ɑ-stimulated procoagulant activity and the time difference between unstimulated and TNF-ɑ-stimulated procoagulant activity, independently on blood cell counts. Reflecting blood procoagulant activity potential, the coagulation time is calculated as the time taken for recalcified blood to clot and is considered to be a sensitive marker of the potential clot formation and CVD risk [7]. This is because thrombus formation depends upon the procoagulant and inflammatory potential of blood cells, including monocytes, granulocytes, platelets, endothelial cells, and plasma vesicles [11]. TNF-ɑ is specifically implicated in inflammation-related thrombosis by promoting extrinsic coagulation activation. This is achieved by inducing tissue factor expression on the leukocyte surface, downregulating natural anticoagulants (protein C and heparin–antithrombin pathways) as well as thrombomodulin and the endothelial protein C receptor, while increasing platelet production, thereby enhancing thrombin formation [12].

On the other hand, *ZBTB12* Factor 1 hypomethylation is associated with higher total WBC and granulocyte counts already having been associated with higher CVD risk and mortality [8, 9]. Neutrophils, the largest part of granulocyte population, are also involved in the formation of neutrophil extracellular traps (NETs), known to play a role in thrombus formation [13]. Both white and red blood cells contribute to the activation of coagulation and to thrombin formation also through the action of their extracellular vesicles (EVs), additional mediators of inflammation [14]. These results suggest a second potential role of *ZBTB12* in affecting myelopoiesis.

*ZBTB12* expression could be regulated through the binding of myelopoiesis and hematopoiesis-specific TFs, also influenced by DNA methylation [15]. In line with this hypothesis, our data on *ZBTB12* TF binding site prediction showed that the *ZBTB12* CpG units 1, 3–4, and 11 are predicted to be bound by PAX-5 and p53 (units 1 and 3–4), known to be involved in hematopoiesis and B cell differentiation [16] and cell cycle arrest required for terminal myelopoiesis [17, 18], and by E2F-1 (units 3–4 and 11), with a pro-apoptotic role in hematopoiesis [19] (Fig. 4). Supporting this hypothesis, *ZBTB12* expression in blood cells is indeed variable across cell types and differentiation stages (data from the BLUEPRINT Consortium [20], https://blueprint.haem.cam.ac.uk/mRNA). ZBTB12, as all ZBTB proteins, could also in turn bind myelopoiesis-related genes, acting as a TF, thanks to its predicted ability of binding methylcytosine (5mC) and/or oxidized methylcytosine (oxi-mCs)-rich DNA sequences, target sequences for Zn fingers [3]. DNA methylation is a known predictor of cell specification throughout the human hematopoietic lineage [21], and other ZBTB proteins are already described to be specifically involved in granulopoiesis [22] and myeloid development in general [23].

## Conclusion

Our data indicate that *ZBTB12* hypomethylation (of both Factor 1 and Factor 2) that was previously associated with MI risk [5] is linked to shorter TNF-ɑ-stimulated whole blood coagulation time and increased WBC and granulocyte counts. This hitherto undescribed association with blood parameters, known to be implicated in CVD [7–9], further support the hypothesis of a link between *ZBTB12* methylation and CVD risk. Future experimental studies should focus on the specific molecular mechanism(s) of this zinc finger protein in blood cell proliferation, maturation, and activity and its possible role in human cardiovascular disorders.

## Methods

### Study population

Moli-family is a family-based study which aimed to investigate the role of inflammation-mediated activation of hemostasis in CVD risk [6]. A total of 754 subjects (≥ 15 years old) were recruited from 54 extended pedigrees (23 families with and 31 control families without personal or familial history of early-onset MI). All participants were relatives of index subjects enrolled in the Moli-sani cohort study [24], which recruited 24,325 subjects randomly selected from civil registries of the Molise Region, Southern Italy, between 2005 and 2010.

In all subjects, a complete medical history and information about smoking and alcohol-drinking habits were obtained via a structured questionnaire. Height, body weight, and blood pressure were measured as described in [6, 25, 26].

### Blood sample collection and blood functional tests

Biochemical analyses were performed in the centralized Moli-sani laboratory. Blood samples were obtained between 07:00 and 09:00 from participants who had fasted overnight and had refrained from smoking for at least 6 h. Hematological cytometric analyses were performed by the same cell counter (Coulter HMX, Beckman Coulter, IL Milan, Italy), within 1 h from venipuncture. Platelet–leukocyte conjugates, platelet P-selectin, leukocyte CD11b, and L-selectin expression were measured in whole blood for the Moli-family participants, as described [27].

Whole blood procoagulant activity was measured by the coagulation time. Whole blood was incubated for 2 h at 37 °C with or without tumor necrosis factor (TNF)-α (100 ng/ml). The optimal agonist concentration was previously selected on the basis of dose-response curves (not shown). At the end of incubation, whole blood coagulation time (i.e., the time taken for recalcified blood to clot) was assessed by a one-stage clotting time. Briefly, 200-μL whole blood were mixed with 100 μL 25 mM CaCl_2_, and the time to clot formation was recorded (seconds) [28].

### DNA extraction and methylation analysis

Buffy coats of peripheral blood cells were isolated from whole blood samples collected in sodium citrate EDTA tubes and centrifuged at 3000 rpm for 20 min at RT. DNA extraction was done using a silica matrix-based method as described [29]. Of the 754 Moli-family participants, 623 had good quality DNA samples to perform the methylation analysis. We measured *ZBTB12* methylation using the Sequenom EpiTYPER MassARRAY (Agena) platform [15]. Details of the *ZBTB12* region studied (chr6: 31899847-31900326, GRCh38/hg38 Assembly) are reported by Guarrera and colleagues [5]. Bisulfite treatment was conducted on 1 μg of genomic DNA using the MethylDetector kit (Active Motif) as described [15]. All PCR amplifications were performed in duplicate. For the CpG-specific analysis, data were discarded when the duplicate measurements had a standard deviation (SD) ≥ 5% [15, 30, 31]. Sequenom peaks with reference intensity > 2 and overlapping units were excluded from the analysis [15, 30, 31]. To exclude possible intra-plate differences, a sample of K562 DNA was carried on in each plate as an internal control.

Of the 20 CpG units included in the *ZBTB12* region studied [5] (CGI1 in Fig. 1), we excluded the ones having more than 15% of missing values in the Moli-family cohort, leading to a total of 13 CpG (Table 1).

### Statistical analysis

Statistical analyses were performed using SAS/STAT software (Version 9.4 for Windows©2009. SAS Institute Inc. and SAS are registered trademarks of SAS Institute Inc., Cary, NC, USA). Mean and SD were computed for continuous variables and frequencies for categorical variables. All continuous variables, including methylation data, were also transformed to *z*-scores (mean = 0; SD = 1).

Correlation analysis among *ZBTB12* CpG units was initially conducted to discover the architecture of relationships among the methylation units studied. Then, a PCA was conducted with the aim of identifying common underlying patterns that could explain the largest part of common variance in methylation across units. PCA was conducted including the 342 individuals having all the 13 *ZBTB12* CpG units successfully measured. Criteria for factor selection were eigenvalue > 1.0 as revealed by the scree test, and the interpretability of the final solution. This resulted in the identification of two main factors (Table 1), which were transformed by the orthogonal varimax rotation to keep independent latent variables for subsequent analysis [32], and then standardized. We characterized the factors using the *ZBTB12* methylation sites with an absolute factor loading > 0.40. Each subject received a factor score, calculated by summing the observed methylation site values, each weighted by factor loadings.

We first studied the association between *ZBTB12* methylation factors and CVD risk factors (unstimulated and TNFɑ-stimulated coagulation time along with the resulting unstimulated–stimulated delta difference, platelet–monocyte and platelet–PMN aggregates, and blood cell counts) (Table 2), in linear mixed effect regression models adjusted for age, sex (fixed effects), and family stratification (random effect) to account for the family structure of the Moli-family cohort.

Similarly, linear mixed effect regression models were used to assess the relation of blood parameters related to CVD risk, with *ZBTB12* methylation patterns (Factor 1 and Factor 2) and single CpG units. Age, gender, smoking (never-, ex-, and current smokers), and variables significantly associated with both methylation factors and specific phenotypes at *p* < 0.1 were treated as fixed effects, while family stratification was treated as a random effect. A false discovery rate (FDR) method (Benjamini–Hochberg) was used to adjust *p* values for multiple testing. A *p* value (pFDR) < 0.05 was considered as statistically significant. DNA methylation is cell specific and might be different among the leukocyte sub-populations, leading to false positive findings when an appropriate correction for cell count is not performed [33]. *ZBTB12* was not identified as a locus with leukocyte-specific DNA methylation levels [33]. Therefore, in our analysis, we did not correct for WBC counts.

### Prediction of *ZBTB12* DNA binding factors

To detect potential regulatory effects of methylation at the CpG sites investigated, we searched for *ZBTB12* putative binding sites, by using the PROMO software [34]. More specifically, we included in our query the regions surrounding each of the CpG sites which were found as associated with blood cell parameters in previous analyses. This allows to construct weight matrices from known binding sites extracted from the TRANSFAC DNA binding site library (version 8.3), which contains the largest available collection of DNA binding sites in eukaryotes [35, 36]. The full *ZBTB12* region analyzed in the methylation study (chr6: 31899847-31900326, GRCh38/hg38 Assembly) was used as DNA sequence bait in the search. The prediction was made by focusing only on the human species and transcription factors, setting the minimum sequence similarity threshold for TF binding detection to 85%.

## Additional file

Additional file 1: Putative transcription factor (TF) binding analysis of the blood cell count specific CpG units. Transcription factor predicted to bind to blood cell count specific CpG units. (DOCX 13 kb)

## Abbreviations

CVD: Cardiovascular disease
EVs: Extracellular vesicles
FDR: False discovery rate
MBPs: Methyl-CpG-binding proteins
MI: Myocardial infarction
NETs: Neutrophil extracellular traps
PCA: Principal component analysis
SD: Standard deviation
TF: Transcription factor
TNF: Tumor necrosis factor
WBC: White blood cells
ZBTB12: Zinc finger and BTB domain-containing protein 12
