# Supplementary material for: ZBTB12 DNA methylation is associated with coagulation- and inflammation-related blood cell parameters: findings from the Moli-family cohort
Source: Clin Epigenetics. 2019 May 10;11:74. doi: 10.1186/s13148-019-0665-6 (PMC6511189; doi:10.1186/s13148-019-0665-6)
Supplement: Supplementary file 1 — Putative transcription factor (TF) binding analysis of the blood cell count specific CpG units. Transcription factor predicted to bind to blood cell count specific CpG units. (DOCX 13 kb) [file 13148_2019_665_MOESM1_ESM.docx]

**Additional file 1. Putative Transcription Factor (TF) binding analysis of the blood cell count specific CpG units**

| **CpG** | **TF** |
| --- | --- |
| **n.** |  |
| **3-4** | XBP-1 |
|  | GCF |
|  | PAX-5 |
|  | p53 |
|  | E2F-1 |
| **26** | ENKTF-1 |
|  | HNF-1C |
| **1** | PAX-5 |
|  | p53 |
|  | TFII-I |
|  | c-Ets-1 |
|  | STAT4 |
|  | Elk-1 |
| **11** | E2F-1 |
| **5** | GR-alpha |
| **27** | HNF-1C |
|  | HNF-1A |
|  | FOXP3 |
|  | HNF-1B |
|  | ATF-1 |
|  | TFII-I |
